# Supplementary material for: Analysis of Bacteriohopanoids from Thermophilic Bacteria by Liquid Chromatography–Mass Spectrometry
Source: Microorganisms. 2021 Sep 30;9(10):2062. doi: 10.3390/microorganisms9102062 (PMC8537080; doi:10.3390/microorganisms9102062)
Supplement: Supplementary file 1 [file microorganisms-09-02062-s001.zip › microorganisms-1400911-supplementary.pdf]

Analysis of Bacteriohopanoids from Thermophilic Bacteria by Liquid Chromatography-Mass Spectrometry

Irena Kolouchová [1], Elizaveta Timkina [1], Olga Maťátková [1], Lucie Kyselová [2] and Tomáš Řezanka[3,*]

[1] Department of Biotechnology, Faculty of Food and Biochemical Technology, University of Chemistry and Technology Prague, Technická 5, 16628 Prague, Czech Republic; irena.kolouchova@vscht.cz
[2] Research Institute of Brewing and Malting, Lípová 511, 120 44 Prague, Czech Republic; kyselova@beerresearch.cz
[3] Institute of Microbiology, the Czech Academy of Sciences, 142 20 Prague 4, Vídeňská1083, Czech Republic; rezanka@biomed.cas.cz
* Correspondence: rezanka@biomed.cas.cz

Table S1. Selected microorganisms analysed for the presence of bacteriohopanepolyols.

Current Name of Microorganism	Alternative Approved Name	Number of SHC Records	Example of GenBank Accession Number	References
**BACTERIA**				
Alicyclobacillus acidocaldarius	–	53	WP_014465455.1	[1–5]
strain 104-1A	ATCC 27009		–	[3,4]
Bacillus subtilis (spores)	–	>100	AAB84441.1	e.g. [6]
strain TB10	–	1	AHA77980.1	[6]
Blastochloris viridis	*Rhodopseudomonas viridis*	0	CUU42009.1 squalene synthase	[7]
Bradyrhizobium japonicum	*Rhizobium japonicum*	19	CAA60250.1	[8,9]
Ca. Brocadia sp.	–	18	QQR66062.1	[10]
Burkholderia cepacia	*Pseudomonas cepacia*	>100	KML22412.1	[11]
strain WH 8501	–	1	EAM53094.1	[12]
Desulfovibrio bastinii	*Maridesulfovibrio bastinii*	1	WP_034633519.1	[13]
strain DSM 16055	–	1	WP_034633519.1	[13]
Frankia sp.	–		ABW14125.1	[10]
Ea1-12	DSM 107422	3	WP_112105706.1	[10]
Frateuria aurantia	–	2	AFC87535.1<br>AFC85227.1	[14]
strain DSM 6220	–	2	AFC87535.1<br>AFC85227.1	[14]
Geobacter metallireducens	–	6	EHP88049.1	[15]
strain DSMZ 7210	GS-15	2	ABB33038.1<br>ABB30662.1	[15]
Geobacter sulfurreducens		11	WP_045667657.1	[15]
Komagataeibacter xylinus	*Acetobacter aceti* subsp. *xylinus*	15	WP_159262470.1	[10,16]
strain R-2277	–	0	–	[10]
Methylacidiphilum fumariolicum	–	5	WP_009061034.1	[17]
strain Sol V	–	1	CCG92847.1	[17]
Methylobacterium fujisawaense	–	0	WP_182591194.1 squalene synthase	[18]
Methylobacterium organophilum	–	5	PVY93823.1	[19]
Methylocella palustris	–	0	–	[10,17]
Methylococcus capsulatus	–	5	WP_010960137.1	[20–24]
strain NCBI 11132	–	0	–	[20]
strain ATCC 33009	–	0	–	[21]
Methylomirabilis oxyfera	–	2	CBE67168.1<br>KAB2961492.1	[25]
Methylomonas methanica	–	5	OAI06767.1	[20,21]
strain NCBI 11130	–	1	OAI06767.1	[20]
strain ATCC 35067	–	0	–	[21]
Methylophilus methylotrophus	–	0	WP_018985602.1 squalene synthase	[21]
Methylosinus trichosporium	–	1	ATQ69413.1	[21,23]
OB3b	–	1	ATQ69413.1	[21]
Methylovulum psychrotolerans	–	9	WP_103975581.1	[26]
strain Sph1^{T}	LMG 29227	2	POZ51441.1<br>POZ50074.1	[26]
strain OZ2	–	0	–	[26]
strain Sph56	–	0	MH701868.1	[26]

			partial sequence	
Nitrosomonas europaea	–	1	WP_011111759.1	[27]
Paenibacillus arachidis	–	0	–	[28]
Rhodobacter capsulatus	*Rhodopseudomonas capsulata*	0	P17056.1<br>phytoene synthase	[7]
Rhodoblastus acidophilus	*Rhodopseudomonas acidophila*	5	WP_155446705.1	[29,30]
strain DSM 145	–	0	–	[29,30]
Rhodomicrobium vannielii	–	3	WP_210336511.1	[31]
strain ATCC 17100	–	1	ADP72221.1	[31]
Rhodopseudomonas palustris	–	36	WP_047307546.1	[16,29,32–35]
strain DSM 139	–	0	–	[29]
strain TIE1	–	1	ACF02757.1	[32–35]
strain DSM-123	–	0	–	[34]
strain CGA009	–	1	WP_011159278.1	[34]
strain BisA53	–	1	ABJ08391.1	[34]
strain BisB5	–	1	ABE40796.1	[34]
strain BisB18	–	1	ABD87279.1	[34]
strain HaA2	–	1	ABD06434.1	[34]
Rubrivivax gelatinosus	*Rhodopseudomonas gelatinosa*	0	WP_200222390.1<br>squalene synthase	[7]
Salinicoccus halitifaciens	–	0	–	[36]
Ca. Scalindua brodeae	*Ca.* Scalindua sorokinii	1	KHE93133.1	[10]
Streptomyces coelicolor	–	3	QFI46551.1	[37]
strain A3(2)	–	2	QFI46551.1<br>CAB39697.1	[37]
Streptomyces peucetius	–	1	ATW51727.1	[38]
strain *S. peucetius* subsp. *caesius* ATCC 27952	–	2	ACA52082.1<br>ATW51727.1	[38]
Streptomyces scabiei	–	3	WP_059080096.1	[39]
strain 87-22	–	1	CBG68454.1<br>(putative)	[39]
Streptomyces venezuelae	*Streptomyces venezuelensis*	21	WP_150501443	–
Thiocapsa rosea	*Amoebobacter roseus*	0	–	[11]
Zymomonas mobilis	–	46	WP_038259183.1	[22]
Zymomonas mobilis subsp. *mobilis*	–	21	ACV76194.1	[40]
strain ATCC 29191	–	2	AFN57413.1<br>AFN56323.1	[40]
**CYANOBACTERIA - marine**				
Crocosphaera watsonii	–	9	WP_021835865.1	[12,41]
WH8501	–	1	EAM53094.1	[41]
Synechococcus sp.	–	10	AFY75324.1	[41]
strains PCC 6907, PCC 6714, WH8102	–	0	–	[41]
Trichodesmium erythraeum	–	2	WP_011610552.1	[41]
strain IMS-101	–	1	ABG50159.1	[41]
CYANOBACTERIA – non-marine				
*Anabena cylindrica**	–	0	–	[41]
*Anacystis montana**	–	0	–	[41]
Calothrix sp.	–	23	WP_096687654.1	[41]
Chlorogloeopsis sp.	–	0	–	[41,42]
Chlorogloeopsis fritschii	–	2	WP_016878379.1<br>RUR72374.1	[41]

Chroococcidiopsis sp.	–	1	WP_169244124.1	[41]
Cyanothece sp.	–	2	WP_107670655.1 WP_012628954.1	[41]
Gleocapsa sp.	–	3	ELS00147.1	[41]
Microcystis sp.	–	> 100	WP_002732984.1	[41]
strains CCAP 1405/3, CCAP 1450/13, 110	–	0	–	[41]
Microcystis aeruginosa	–	100	WP_052276441.1	[41]
strain PCC 7808	–	0	–	[41]
Nostoc sp.	*Amorphonostoc*	96	AFY41090.1	[41]
strain PCC 6720	–	0	–	[41]
Nostoc muscorum	*Desmonostoc muscorum*	2	WP_193917519.1 WP_190962091	[41]
strains B 1452-12b, CCAP 1453/12	–	0	–	[41]
Oscillatoria amphigranulata	*Pseudanabaena amphigranulata*	0	–	[41]
Phormidium sp.	–		WP_190625411.1	[41]
strain OSS4	OLV, CYX	0	–	[41]
strain RCG	PR11	0	–	[41]
strain RCO	–	0	–	[41]
Phormidium luridum	*Drouetiella lurida*	0	–	[41]
Prochlorothrix hollandica	–	1	WP_017714382.1	[41,42]
strain CCAP 1490/1	–	0	–	[41]
Synechocystis sp.	–	10	WP_028946973.1	[41]
PCC 6714	–	1	AIE72536.1	[41]
PCC 6803	–	8	QWO81910.1	[41]

SHC – squalene-hopene cyclase

* no record at all in the NCBI database

Table S2. List of the cultivated bacterial isolates from four springs of Carlsbad (Karlovy Vary), Czech Republic and ten strains obtained from the Czech Collection of Microorganisms, Brno, Czech Republic.

Bacterium	Cultiv. Temp.	Reference
Geobacillus stearothermophilus CCM 2062 (no information about source)	70	[43]
Geobacillus stearothermophilus CCM 5965 (Evaporated milk)	70	[43]
***Geobacillus stearothermophilus* ST-YPD (spring Štěpánka)** [a]	**58**	[44]
***Geobacillus stearothermophilus* VR-1 (spring Vřídlo)** [a]	**58**	[44]
Geobacillus stearothermophilus CCM 2062	55	[45]
Meiothermus ruber CCM 4212 (Thermal pools, Hveragherti, Iceland)	70	[43]
Meiothermus ruber CCM 4212	65	[46]
Meiothermus ruber CCM 4212	55	[45]
Alicyclobacillus acidoterrestris CCM 4660 (Apple-grape-raspberry juice)	70	[43]
Alicyclobacillus acidoterrestris CCM 4660	45	[47]
Thermus aquaticus CCM 3488 (Thermally polluted river near Brussels, Belgium)	70	[43]
Thermus aquaticus CCM 3488,	65	[46]
***Geobacillus kaustophilus* ML-1 (spring Mlýnský)** [a]	**58**	[44]
***Brevibacillus agri* SA-1 (spring Sadový)** [a]	**42**	[44]

[a] The strains obtained from the thermal springs in Carlsbad, Czech Republic.

Table S3. List of the cultivated bacterial isolates from four springs of Carlsbad (Karlovy Vary), Czech Republic.

Isolate	Hot spring	Cultivation	GenBank Accession
Brevibacillus agri SA-1	Sadový	42	MT251434
Geobacillus kaustophilus ML-1	Mlýnský	58	MT251494
Geobacillus stearothermophilus ST-YPD	Štěpánka	58	MT251887
Geobacillus stearothermophilus VR-1	Vřídlo	58	MT251886

Table S4. Separation and identification of hopanoids from thermophilic bacteria by RP-LC/MS-ESI.

Abbreviation	Chemical Formula	*m/z*	Abundance (%)	tR (min)	Structure
**1a**	$[C_{47}H_{76}NO_{11}]^+$	830.5413	42	23.10	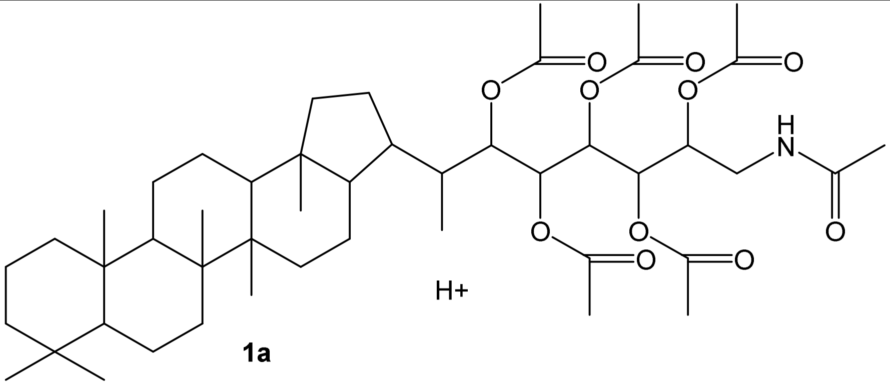
**1b**	$[C_{59}H_{100}NO_{11}]^+$	998.7294	8	29.38	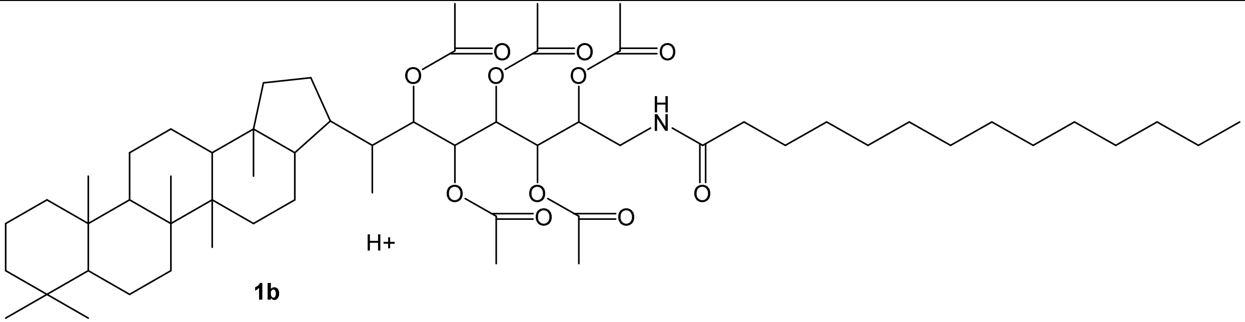
**1c**	$[C_{60}H_{102}NO_{11}]^+$	1012.7447	34	35.06+ 35.45	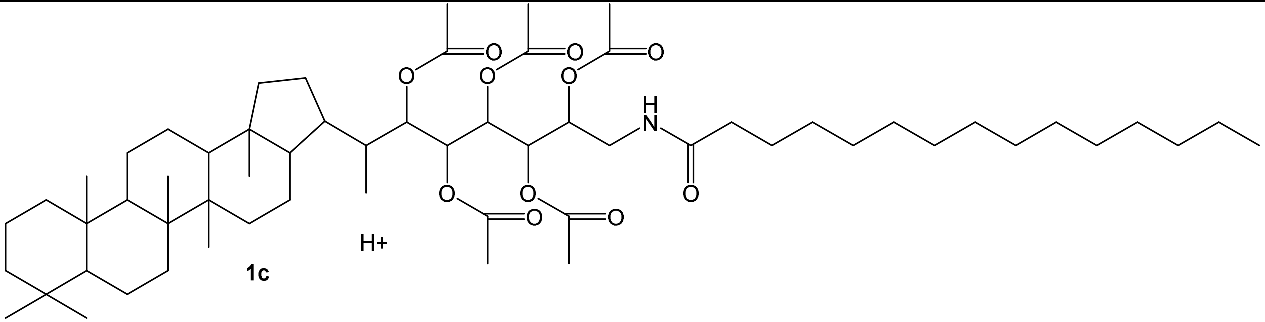

**1d**	$[C_{61}H_{104}NO_{11}]^+$	1026.7604	19	39.28	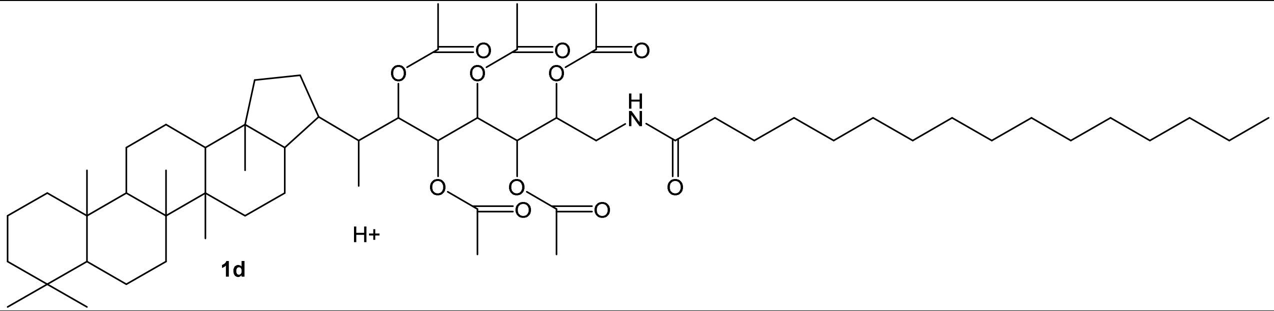
**1e**	$[C_{62}H_{106}NO_{11}]^+$	1040.7760	22	42.67+<br>43.03	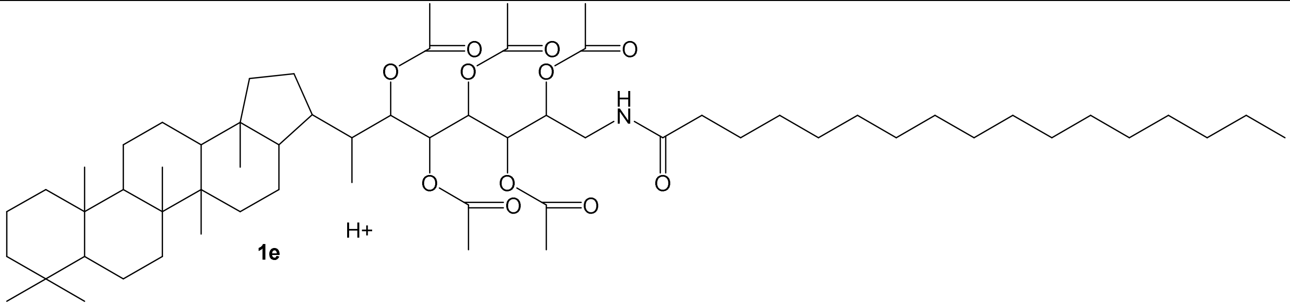
**2a**	$[C_{45}H_{74}NO_{9}]^+$	772.5358	21	26.17	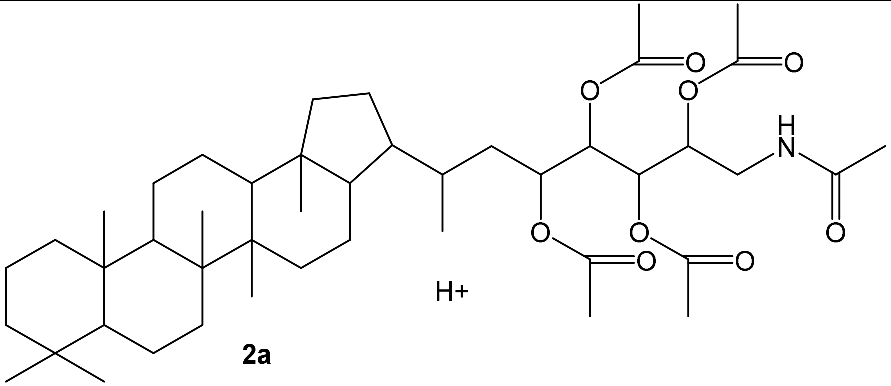
**2b**	$[C_{57}H_{98}NO_{9}]^+$	940.7236	5	33.32	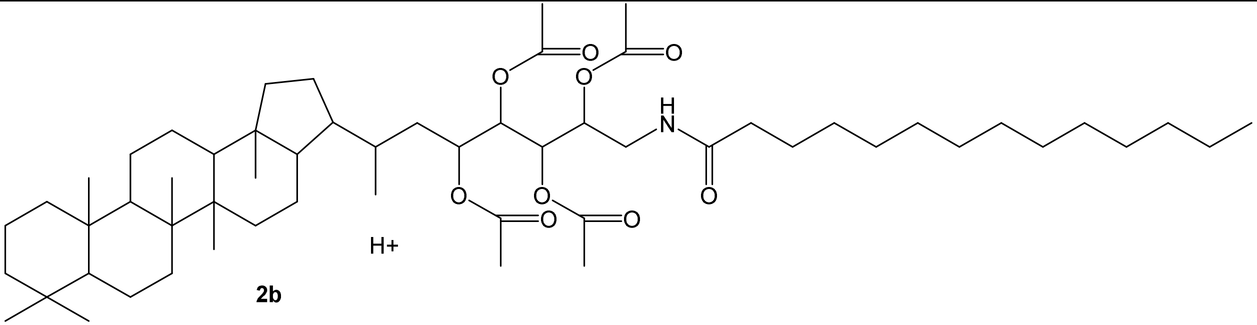

**2c**	$[C_{58}H_{100}NO_9]^+$	954.7393	17	38.11+ 38.63	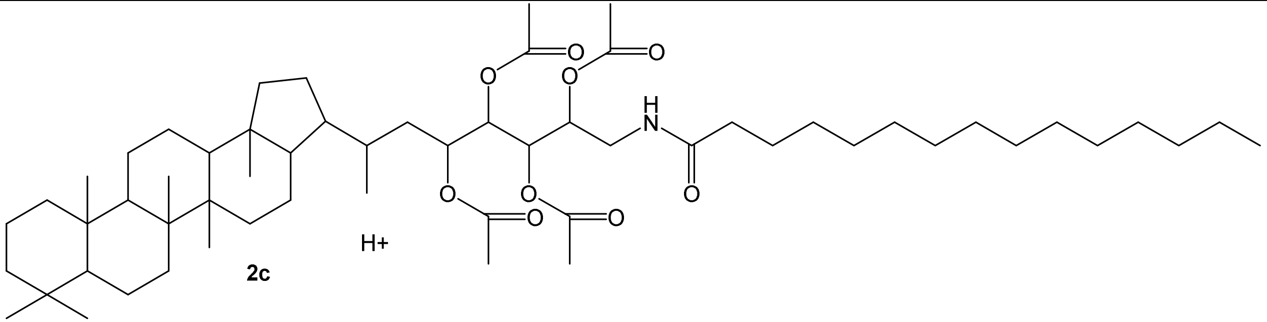
**2d**	$[C_{59}H_{102}NO_9]^+$	968.7549	11	41.45	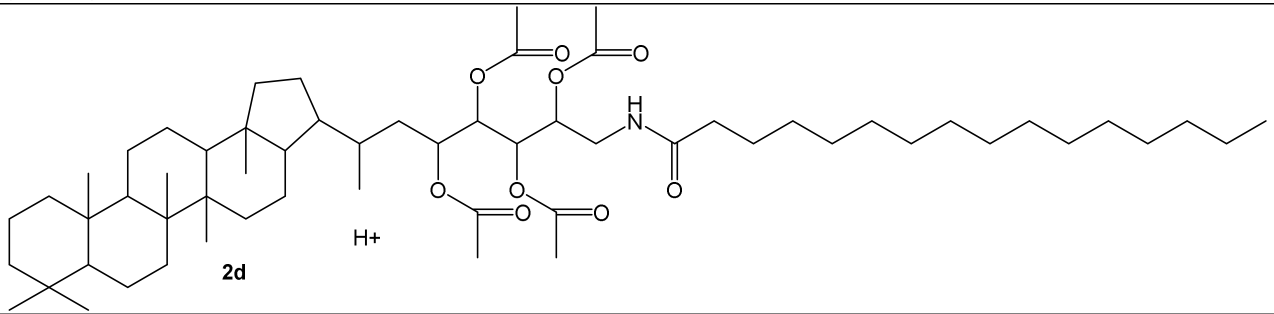
**2e**	$[C_{60}H_{104}NO_9]^+$	982.7706	14	45.16	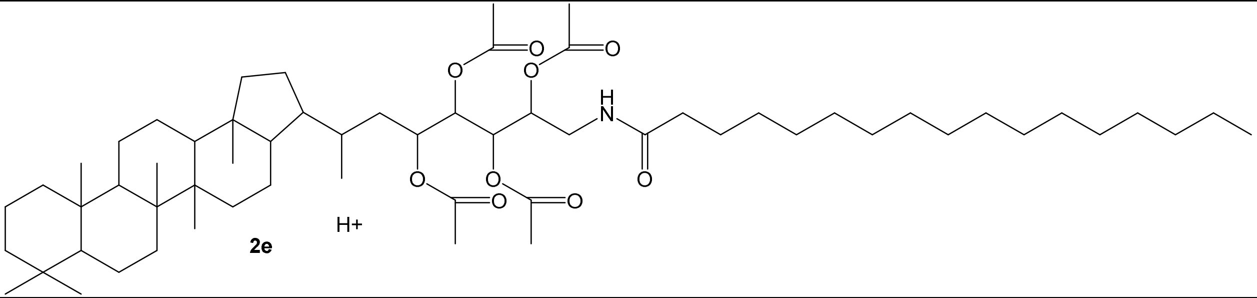
**3a**	$[C_{43}H_{72}NO_7]^+$	714.5303	23	34.24	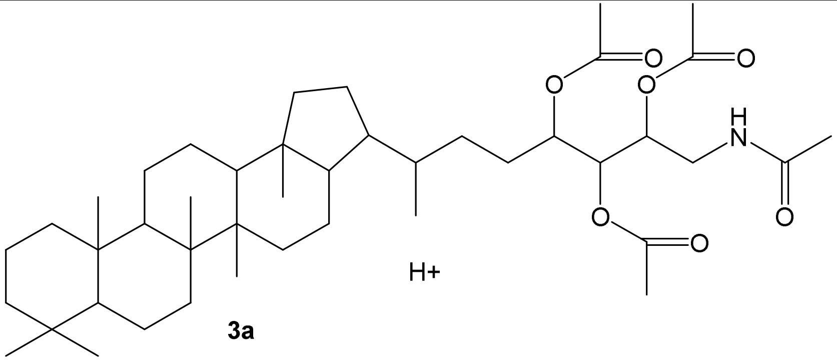

**3b**	$[C_{55}H_{96}NO_7]^+$	882.7181	5	36.89	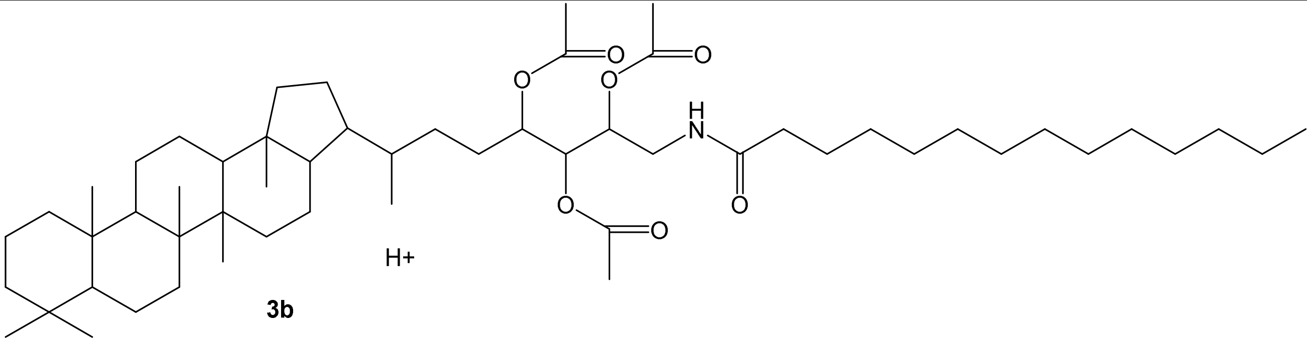
**3c**	$[C_{56}H_{98}NO_7]^+$	896.7338	45	42.12	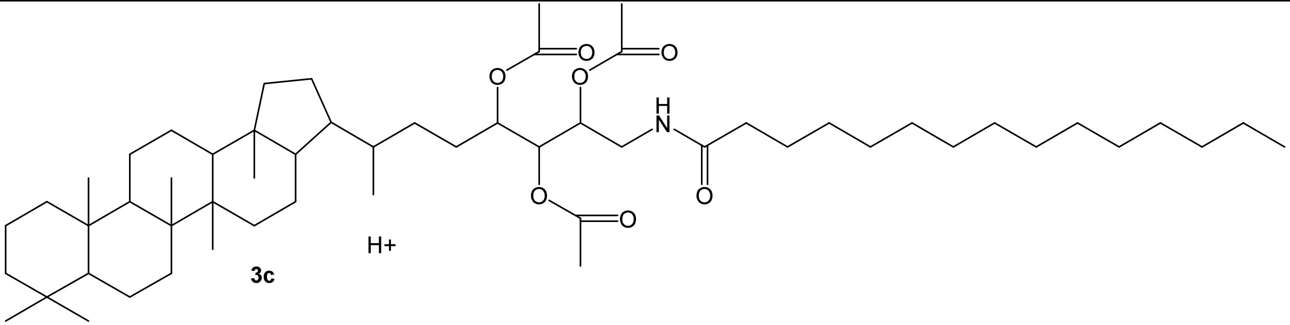
**3d**	$[C_{57}H_{100}NO_7]^+$	910.7494	33	46.65	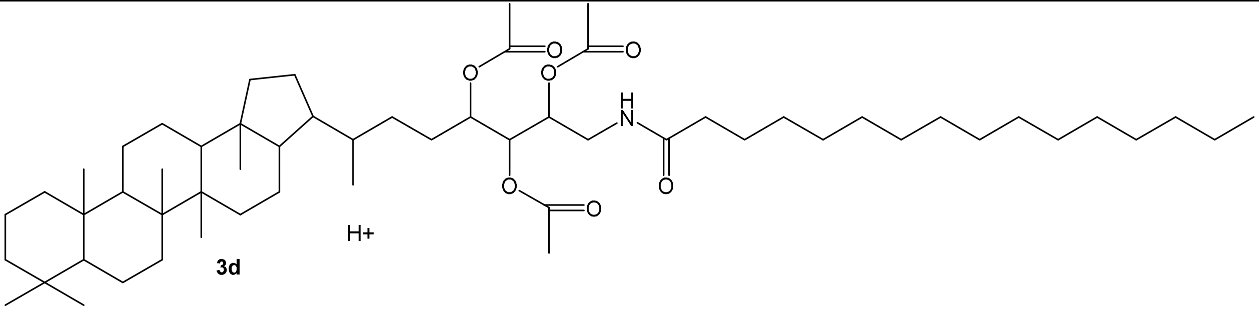
**3e**	$[C_{58}H_{102}NO_7]^+$	924.7651	24	49.74	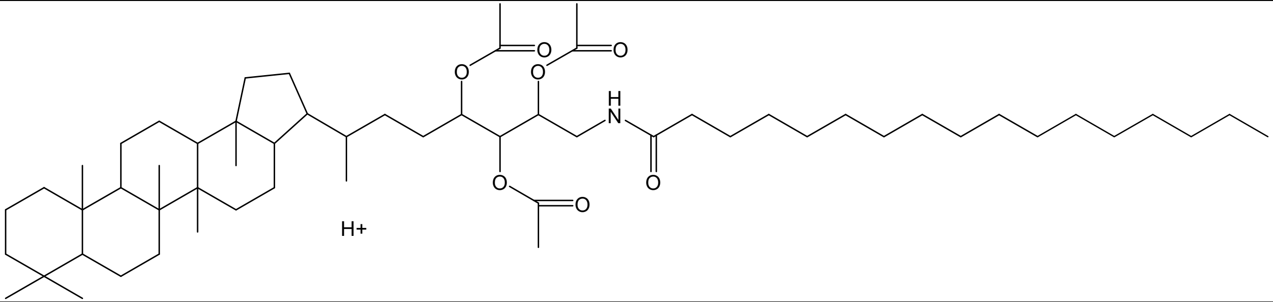

**4**	$[C_{46}H_{70}N_5O_6]^+$	788.5321	18	32.13	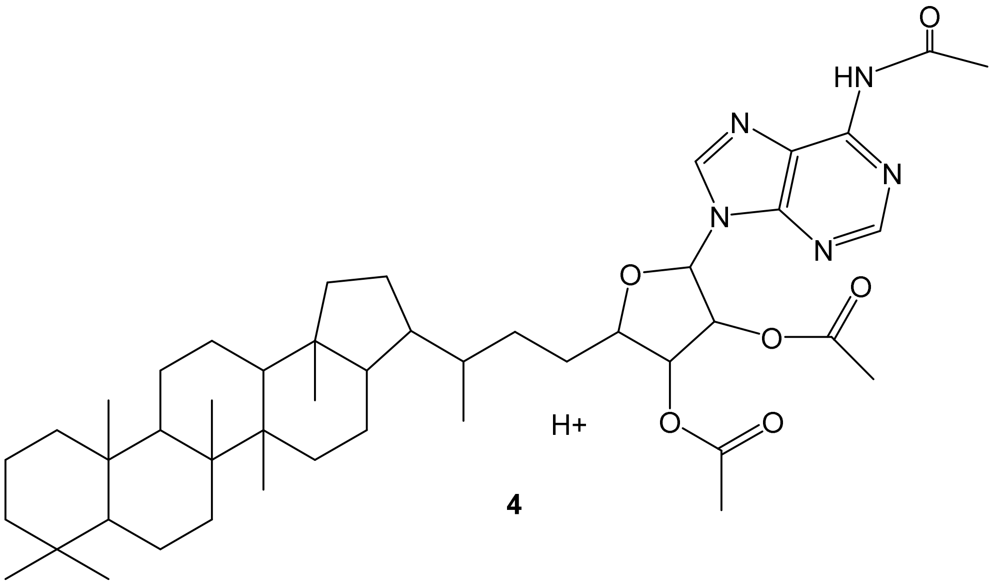 **4**
**5**	$[C_{43}H_{71}O_8]^+$	715.5143	100	30.14	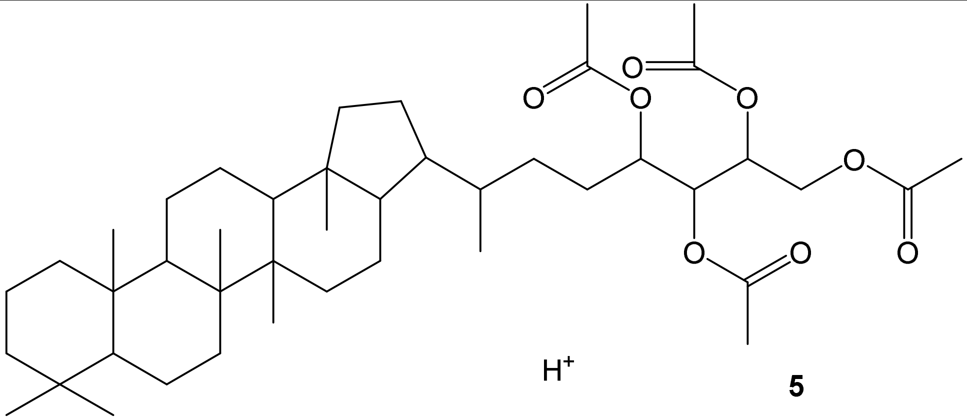 **5**

**6**	$[C_{55}H_{88}NO_{15}]^+$	1002.6151	9	28.54	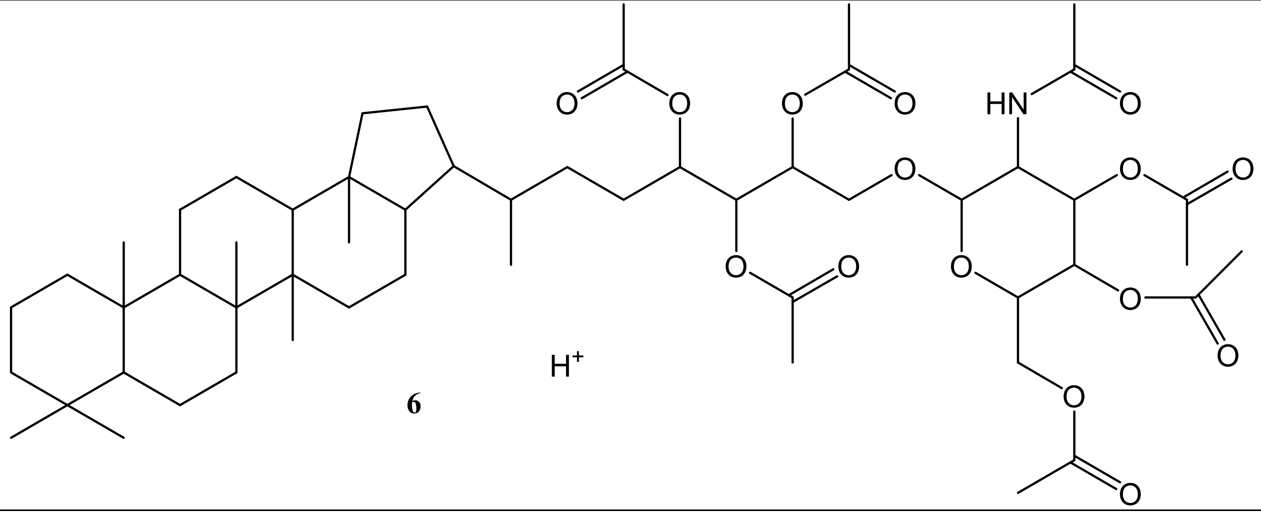<br>**6**

Table S5. Occurrence of hopanoid C-2 methylase and/or hopanoid C-3 methylase in analyzed thermophilic bacteria. In the table *hpnP* stands for hopanoid C-2 methylase gene and *hpnP* for hopanoid C-3 methylase. Sequencies used for analysis were as follows: B3QHD1 Hopanoid C-2 methylase from *Rhodopseudomonas palustris* (strain TIE-1) and Q60AV6 Hopanoid C-3 methylase from *Methylococcus capsulatus* (strain ATCC 33009).

	Alicyclobacillus acidoterrestris		***Brevibacillus agri***		***Geobacillus kaustophilus***		***Geobacillus stearothermophilus***		***Geobacillus thermoglucosidasius***	
**Enzyme**	*hpnP*	*hpnR*	*hpnP*	*hpnR*	*hpnP*	*hpnR*	*hpnP*	*hpnR*	*hpnP*	*hpnR*
**Detected protein with the highest similarity**	B12-binding domain-containing radical SAM protein	B12-binding domain-containing radical SAM protein	B12-binding domain-containing radical SAM protein	B12-binding domain-containing radical SAM protein	Mg-protoporphyrin IX monomethyl ester oxidative cyclase	Mg-protoporphyrin IX monomethyl ester oxidative cyclase	B12-binding domain-containing radical SAM protein	B12-binding domain-containing radical SAM protein	B12-binding domain-containing radical SAM protein	B12-binding domain-containing radical SAM protein
**Similarity percentage**	61.2 %	84.3 %	48.5 %	63.5 %	48.9 %	65.9 %	55.5 %	92.0 %	50.4 %	64.7 %
**ID**	EPZ45238.1	EPZ45238.1	ELK42704.1	QAV14985.1<br>RNB47569.1<br>GED27563.1	BAD75227.1	BAD75227.1	KZM55362.1<br>QOR85095.1	ALA71377.1	RDE27903.1<br>WP_003252170.1<br>GCD83585.1	GCD83585.1<br>RDE27903.1<br>ALF09928.1

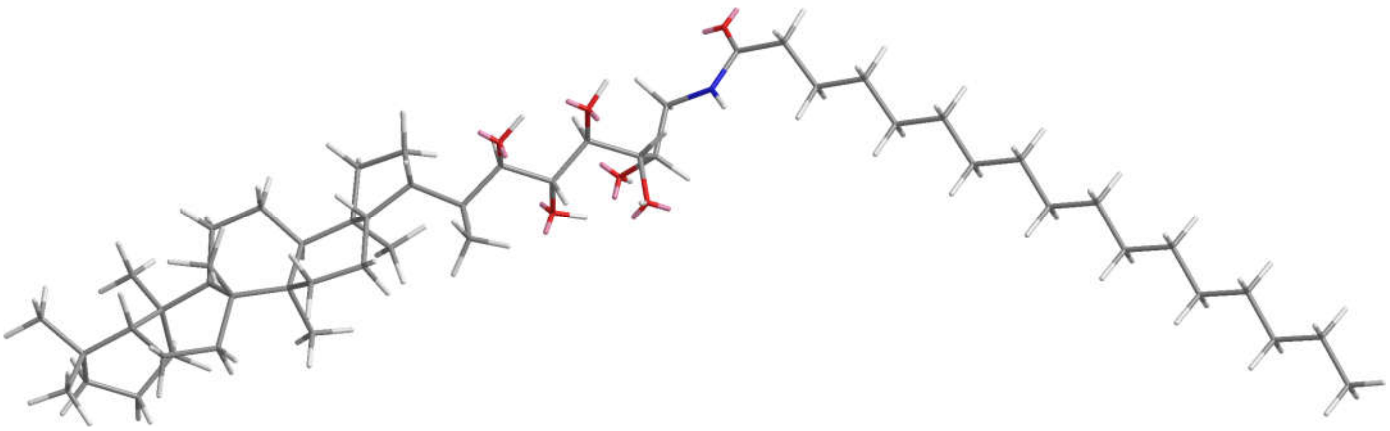

Figure S1. Model of *N*-acyl derivative of bacteriohopanepentol.

References

1. Langworthy, T.; Mayberry, W. 1,2,3,4-Tetrahydroxy Pentane-Substituted Pentacyclic Triterpene from *Bacillus acidocaldarius*. *Biochim. Biophys. Acta* **1976**, *431*, 570–577, doi:10.1016/0005-2760(76)90221-6.
2. Kannenberg, E.; Blume, A.; Geckeler, K.; Poralla, K. Properties of Hopanoids and Phosphatidylcholines Containing Omega-Cyclohexane Fatty-Acid in Monolayer and Liposome Experiments. *Biochim. Biophys. Acta* **1985**, *814*, 179–185, doi:10.1016/0005-2736(85)90434-1.
3. Hippchen, B.; Röll, A.; Poralla, K. Occurrence in Soil of Thermo-Acidophilic Bacilli Possessing ω-Cyclohexane Fatty Acids and Hopanoids. *Arch. Microbiol.* **1981**, *129*, 53–55, doi:10.1007/BF00417180.
4. Poralla, K.; Härtner, T.; Kannenberg, E. Effect of Temperature and pH on the Hopanoid Content of *Bacillus acidocaldarius*. *FEMS Microbiol. Lett.* **1984**, *23*, 253–256, doi:10.1111/j.1574-6968.1984.tb01073.x.
5. Siedenburg, G.; Jendrossek, D. Squalene-Hopene Cyclases. *Appl. Environ. Microbiol.* **2011**, *77*, 3905–3915, doi:10.1128/AEM.00300-11.
6. Kontnik, R.; Bosak, T.; Butcher, R.A.; Brocks, J.J.; Losick, R.; Clardy, J.; Pearson, A. Sporulenes, Heptaprenyl Metabolites from *Bacillus subtilis* Spores. *Org. Lett.* **2008**, *10*, 3551–3554, doi:10.1021/ol801314k.
7. Talbot, H.M.; Rohmer, M.; Farrimond, P. Rapid Structural Elucidation of Composite Bacterial Hopanoids by Atmospheric Pressure Chemical Ionisation Liquid Chromatography/Ion Trap Mass Spectrometry. *Rapid Commun. Mass Spectrom.* **2007**, *21*, 880–892, doi:10.1002/rcm.2911.
8. Perzl, M.; Müller, P.; Poralla, K.; Kannenberg, E.L. Squalene-Hopene Cyclase from *Bradyrhizobium japonicum*: Cloning, Expression, Sequence Analysis and Comparison to Other Triterpenoid Cyclases. *Microbiology* 1997, *143*, 1235–1242.
9. Kannenberg, E.L.; Perzl, M.; Müller, P.; Härtner, T.; Poralla, K. Hopanoid Lipids in *Bradyrhizobium* and Other Plant-Associated Bacteria and Cloning of the *Bradyrhizobium japonicum* Squalene-Hopene Cyclase Gene. *Plant Soil* **1996**, *186*, 107–112, doi:10.1007/BF00035063.
10. Schwartz-Narbonne, R.; Schaeffer, P.; Hopmans, E.C.; Schenesse, M.; Charlton, E.A.; Jones, D.M.; Damste, J.S.S.; Ul Haque, M.F.; Jetten, M.S.M.; Lengger, S.K.; et al. A Unique Bacteriohopanetetrol Stereoisomer of Marine Anammox. *Org. Geochem.* **2020**, *143*, doi:10.1016/j.orggeochem.2020.103994.
11. Rohmer, M.; Bouvier-Nave, P.; Ourisson, G. Distribution of Hopanoid Triterpenes in Prokaryotes. *Microbiology,* 1984, *130*, 1137–1150.
12. Sáenz, J.P.; Waterbury, J.B.; Eglinton, T.I.; Summons, R.E. Hopanoids in Marine Cyanobacteria: Probing Their Phylogenetic Distribution and Biological Role. *Geobiology* **2012**, *10*, 311–319, doi:10.1111/j.1472-4669.2012.00318.x.
13. Blumenberg, M.; Oppermann, B.I.; Guyoneaud, R.; Michaelis, W. Hopanoid Production by *Desulfovibrio bastinii* Isolated from Oilfield Formation Water. *FEMS Microbiol. Lett.* **2009**, *293*, 73–78, doi:10.1111/j.1574-6968.2009.01520.x.
14. Joyeux, C.; Fouchard, S.; Llopiz, P.; Neunlist, S. Influence of the Temperature and the Growth Phase on the Hopanoids and Fatty Acids Content of *Frateuria aurantia* (DSMZ 6220). *FEMS Microbiol. Ecol.* **2004**, *47*, 371–379, doi:10.1016/S0168-6496(03)00302-7.
15. Eickhoff, M.; Birgel, D.; Talbot, H.M.; Peckmann, J.; Kappler, A. Bacteriohopanoid Inventory of *Geobacter sulfurreducens* and *Geobacter metallireducens*. *Org. Geochem.* **2013**, *58*, 107–114, doi:10.1016/j.orggeochem.2013.02.013.

16. Talbot, H.M.; Rohmer, M.; Farrimond, P. Structural Characterisation of Unsaturated Bacterial Hopanoids by Atmospheric Pressure Chemical Ionisation Liquid Chromatography/Ion Trap Mass Spectrometry. *Rapid Commun. Mass Spectrom.* **2007**, *21*, 1613–1622, doi:10.1002/rcm.2997.
17. van Winden, J.F.; Talbot, H.M.; Kip, N.; Reichart, G.-J.; Pol, A.; McNamara, N.P.; Jetten, M.S.M.; Op den Camp, H.J.M.; Sinninghe Damsté, J.S. Bacteriohopanepolyol Signatures as Markers for Methanotrophic Bacteria in Peat Moss. *Geochim. Cosmochim. Acta* **2012**, *77*, 52–61, doi:10.1016/j.gca.2011.10.026.
18. Knani, M.; Corpe, W.; Rohmer, M. Bacterial Hopanoids from Pink-Pigmented Facultative Methylotrophs (PPFMs) and from Green Plant-Surfaces. *Microbiol.-UK* **1994**, *140*, 2755–2759, doi:10.1099/00221287-140-10-2755.
19. Liu, W.; Bodlenner, A.; Rohmer, M. Hemisynthesis of Deuteriated Adenosylhopane and Conversion into Bacteriohopanetetrol by a Cell-Free System from *Methylobacterium organophilum*. *Org. Biomol. Chem.* **2015**, *13*, 3393–3405, doi:10.1039/c4ob02560a.
20. Neunlist, S.; Rohmer, M. Novel Hopanoids from the Methylotrophic Bacteria *Methylococcus capsulatus* and *Methylomonas methanica* - (22S)-35-Aminobacteriohopane-30,31,32,33,34-Pentol and (22s)-35-Amino-3-Beta-Methylbacteriohopane-30,31,32,33,34-Pentol. *Biochem. J.* **1985**, *231*, 635–639, doi:10.1042/bj2310635.
21. Jahnke, L.L.; Summons, R.E.; Hope, J.M.; Marais, D.J.D. Carbon Isotopic Fractionation in Lipids from Methanotrophic Bacteria II: The Effects of Physiology and Environmental Parameters on the Biosynthesis and Isotopic Signatures of Biomarkers. *Geochim. Cosmochim. Acta* **1999**, *63*, 79–93, doi:https://doi.org/10.1016/S0016-7037(98)00270-1.
22. Talbot, H.M.; Squier, A.H.; Keely, B.J.; Farrimond, P. Atmospheric Pressure Chemical Ionisation Reversed-Phase Liquid Chromatography/Ion Trap Mass Spectrometry of Intact Bacteriohopanepolyols. *Rapid Commun. Mass Spectrom.* **2003**, *17*, 728–737, doi:10.1002/rcm.974.
23. Talbot, H.M.; Sidgwick, F.R.; Bischoff, J.; Osborne, K.A.; Rush, D.; Sherry, A.; Spencer-Jones, C.L. Analysis of Non-Derivatised Bacteriohopanepolyols by Ultrahigh-Performance Liquid Chromatography/Tandem Mass Spectrometry. *Rapid Commun. Mass Spectrom.* **2016**, *30*, 2087–2098, doi:https://doi.org/10.1002/rcm.7696.
24. Tippelt, A.; Jahnke, L.; Poralla, K. Squalene-Hopene Cyclase from Methylococcus Capsulatus (Bath): A Bacterium Producing Hopanoids and Steroids. *Biochim. Biophys. Acta* **1998**, *1391*, 223–232, doi:10.1016/S0005-2760(97)00212-9.
25. Kool, D.M.; Talbot, H.M.; Rush, D.; Ettwig, K.; Damsté, J.S.S. Rare Bacteriohopanepolyols as Markers for an Autotrophic, Intra-Aerobic Methanotroph. *Geochim. Cosmochim. Acta* **2014**, *136*, 114–125, doi:https://doi.org/10.1016/j.gca.2014.04.002.
26. Bale, N.J.; Rijpstra, W.I.C.; Sahonero-Canavesi, D.X.; Oshkin, I.Y.; Belova, S.E.; Dedysh, S.N.; Sinninghe Damsté, J.S. Fatty Acid and Hopanoid Adaption to Cold in the Methanotroph *Methylovulum psychrotolerans*. *Front. Microbiol.* **2019**, *10*, 589, doi:10.3389/fmicb.2019.00589.
27. Seemann, M.; Bisseret, P.; Tritz, J.-P.; Hooper, A.B.; Rohmer, M. Novel Bacterial Triterpenoids of the Hopane Series from *Nitrosomonas europaea* and Their Significance for the Formation of the C35 Bacteriohopane Skeleton. *Tetrahedron Lett.* **1999**, *40*, 1681–1684, doi:10.1016/S0040-4039(99)00064-7.
28. Sadaf, K.; Tushar, L.; Nirosha, P.; Podile, A.R.; Sasikala, C.; Ramana, C.V. Paenibacillus Arachidis Sp. Nov., Isolated from Groundnut Seeds. *Int. J. Syst. Evol. Microbiol.* **2016**, *66*, 2923–2928, doi:10.1099/ijsem.0.001124.
29. Neunlist, S.; Bisseret, P.; Rohmer, M. The Hopanoids of the Purple Non-Sulfur Bacteria *Rhodopseudomonasp alustris* and *Rhodopseudomonas acidophila* and the Absolute-Configuration of Bacteriohopanetetrol. *Eur. J. Biochem.* **1988**, *171*, 245–252, doi:10.1111/j.1432-1033.1988.tb13783.x.
30. Neunlist, S.; Rohmer, M. A Novel Hopanoid, 30-(5′-Adenosyl)Hopane, from the Purple Non-Sulfur Bacterium *Rhodopseudomonas acidophila*, with Possible DNA Interactions. *Biochem. J.* **1985**, *228*, 769–771, doi:10.1042/bj2280769.
31. Neunlist, S.; Holst, O.; Rohmer, M. Prokaryotic Triterpenoids - the Hopanoids of the Purple Non-Sulfur Bacterium *Rhodomicrobium vannielii* - an Aminotriol and Its Aminoacyl Derivatives, *N*-Tryptophanyl and *N*-Ornithinyl Aminotriol. *Eur. J. Biochem.* **1985**, *147*, 561–568, doi:10.1111/j.0014-2956.1985.00561.x.
32. Kulkarni, G.; Wu, C.-H.; Newmana, D.K. The General Stress Response Factor EcfG Regulates Expression of the C-2 Hopanoid Methylase HpnP in *Rhodopseudomonas palustris* TIE-1. *J. Bacteriol.* **2013**, *195*, 2490–2498, doi:10.1128/JB.00186-13.
33. Wu, C.-H.; Kong, L.; Bialecka-Fornal, M.; Park, S.; Thompson, A.L.; Kulkarni, G.; Conway, S.J.; Newman, D.K. Quantitative Hopanoid Analysis Enables Robust Pattern Detection and Comparison between Laboratories. *Geobiology* **2015**, *13*, 391–407, doi:10.1111/gbi.12132.

34. Sessions, A.L.; Zhang, L.; Welander, P.V.; Doughty, D.; Summons, R.E.; Newman, D.K. Identification and Quantification of Polyfunctionalized Hopanoids by High Temperature Gas Chromatography-Mass Spectrometry. *Org. Geochem.* **2013**, *56*, 120–130, doi:10.1016/j.orggeochem.2012.12.009.
35. Eickhoff, M.; Birgel, D.; Talbot, H.M.; Peckmann, J.; Kappler, A. Diagenetic Degradation Products of Bacteriohopanepolyols Produced by *Rhodopseudomonas palustris* Strain TIE-1. *Org. Geochem.* **2014**, *68*, 31–38, doi:10.1016/j.orggeochem.2014.01.002.
36. Ramana, C.V.; Srinivas, A.; Subhash, Y.; Tushar, L.; Mukherjee, T.; Kiran, P.U.; Sasikala, C. *Salinicoccus halitifaciens* Sp. Nov., a Novel Bacterium Participating in Halite Formation. *Antonie Van Leeuwenhoek* **2013**, *103*, 885–898, doi:10.1007/s10482-012-9870-4.
37. Liu, W.; Sakr, E.; Schaeffer, P.; Talbot, H.M.; Donisi, J.; Härtner, T.; Kannenberg, E.; Takano, E.; Rohmer, M. Ribosylhopane, a Novel Bacterial Hopanoid, as Precursor of C35 Bacteriohopanepolyols in *Streptomyces coelicolor* A3(2). *ChemBioChem* **2014**, *15*, 2156–2161, doi:https://doi.org/10.1002/cbic.201402261.
38. Ghimire, G.P.; Koirala, N.; Sohng, J.K. Activation of Cryptic Hop Genes from *Streptomyces peucetius* ATCC 27952 Involved in Hopanoid Biosynthesis. *J. Microbiol. Biotechnol.* **2015**, *25*, 658–661, doi:10.4014/jmb.1408.08058.
39. Seipke, R.F.; Loria, R. Hopanoids Are Not Essential for Growth of *Streptomyces scabies* 87-22. *J. Bacteriol.* **2009**, *191*, 5216–5223, doi:10.1128/JB.00390-09.
40. Schmidt, A.; Bringer-Meyer, S.; Poralla, K.; Sahm, H. Effect of Alcohols and Temperature on the Hopanoid Content of *Zymomonas mobilis*. *Appl. Microbiol. Biotechnol.* **1986**, *25*, 32–36, doi:10.1007/BF00252509.
41. Talbot, H.M.; Summons, R.E.; Jahnke, L.L.; Cockell, C.S.; Rohmer, M.; Farrimond, P. Cyanobacterial Bacteriohopanepolyol Signatures from Cultures and Natural Environmental Settings. *Org. Geochem.* **2008**, *39*, 232–263, doi:10.1016/j.orggeochem.2007.08.006.
42. Talbot, H.M.; Summons, R.; Jahnke, L.; Farrimond, P. Characteristic Fragmentation of Bacteriohopanepolyols during Atmospheric Pressure Chemical Ionisation Liquid Chromatography/Ion Trap Mass Spectrometry. *Rapid Commun. Mass Spectrom.* **2003**, *17*, 2788–2796, doi:https://doi.org/10.1002/rcm.1265.
43. Siristova, L.; Melzoch, K.; Rezanka, T. Fatty Acids, Unusual Glycophospholipids and DNA Analyses of Thermophilic Bacteria Isolated from Hot Springs. *Extremophiles* **2009**, *13*, 101–109, doi:10.1007/s00792-008-0202-6.
44. Gharwalova, L.; Palyzova, A.; Maresova, H.; Kolouchova, I.; Kyselova, L.; Rezanka, T. Identification of Homologous Polyprenols from Thermophilic Bacteria. *Microorganisms* **2021**, *9*, 1168, doi:10.3390/microorganisms9061168.
45. Siristova, L.; Luhovy, R.; Sigler, K.; Rezanka, T. Biosynthesis of ω-Alicyclic Fatty Acids Induced by Cyclic Precursors and Change of Membrane Fluidity in Thermophilic Bacteria *Geobacillus stearothermophilus* and *Meiothermus ruber*. *Extremophiles* **2011**, *15*, 423–429, doi:10.1007/s00792-011-0373-4.
46. Rezanka, T.; Siristova, L.; Sigler, K. Rhamnolipid-Producing Thermophilic Bacteria of Species *Thermus* and *Meiothermus*. *Extremophiles* **2011**, *15*, 697–709, doi:10.1007/s00792-011-0400-5.
47. Rezanka, T.; Siristova, L.; Schreiberova, O.; Rezanka, M.; Masak, J.; Melzoch, K.; Sigler, K. Pivalic Acid Acts as a Starter Unit in a Fatty Acid and Antibiotic Biosynthetic Pathway in *Alicyclobacillus*, *Rhodococcus* and *Streptomyces*. *Environ. Microbiol.* **2011**, *13*, 1577–1589, doi:10.1111/j.1462-2920.2011.02465.x.
